# Modifiable Pancreatic Ductal Adenocarcinoma (PDAC) Risk Factors

**DOI:** 10.3390/jcm12134318

**Published:** 2023-06-27

**Authors:** Natalia Michalak, Ewa Małecka-Wojciesko

**Affiliations:** Department of Digestive Tract Diseases, Medical University of Lodz, 90-419 Lodz, Poland; ewa.malecka-panas@umed.lodz.pl

**Keywords:** pancreatic ductal adenocarcinoma, PDAC, risk factors, pancreatic cancer

## Abstract

This study aims to summarize the modifiable risk factors for pancreatic ductal adenocarcinoma (PDAC) that have been known for a long time, as well as information from the most recent reports. As a cancer with a late diagnosis and poor prognosis, accurate analysis of PDAC risk factors is warranted. The incidence of this cancer continues to rise, and the five-year survival rate is the lowest with respect to other tumors. The influence of cigarette smoking, alcohol consumption, and chronic pancreatitis in increasing the risk of pancreatic ductal adenocarcinoma is continually being confirmed. There are also newly emerging reports relating to the impact of lifestyle, including physical activity, the gut and oral microbiome, and hepatotropic viruses. A precise understanding of PDAC risk factors can help to identify groups of high-risk patients, and this may contribute to population awareness and education as well as earlier diagnoses with possible better treatment outcomes.

## 1. Introduction

Pancreatic ductal adenocarcinoma (PDAC) is the most common pancreatic cancer histotype and is known for its high malignancy and late diagnosis [1]. It is characterized by rapid local progression, but also tends to metastasize distantly [2]. According to GLOBCAN (database providing global cancer statistics), pancreatic cancer (PDAC) is the seventh leading cause of cancer death in both sexes with the highest incidence rates throughout Europe, Northern America, and Australia/New Zealand [3]. It is projected to become the second leading cause of cancer deaths in the United States by 2030 [4]. Alarmingly, the incidence of PDAC has been increasing in both sexes in recent years [5]. In this malignancy, the five-year survival rate is only 9% and belongs to the lowest of all cancer types [6]. Due to the constantly limited diagnostic and therapeutic options for PDAC patients, it is essential to analyze modifiable risk factors for this cancer and implement adequate prevention [7].

**Figure 1:**
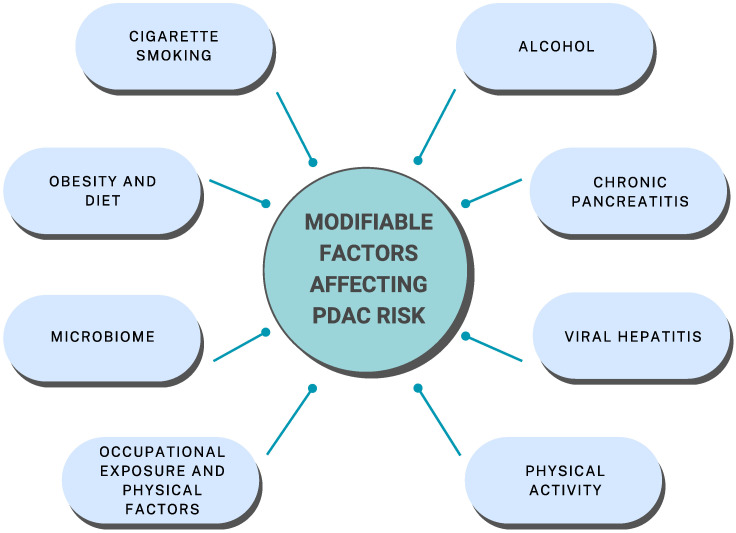
A summary of all the factors discussed in this study that have a positive or negative impact on the risk of PDAC development.

Although PDAC risk factors such as cigarette smoking, alcohol consumption, and chronic pancreatitis have been investigated for years, they are constantly a focus of interest for researchers, but currently, research is in the direction of exploring their underlying mechanisms. On the other hand, recently, the interest of researchers is shifting to factors such as obesity and active lifestyle or the impact of various viruses and bacteria on PDAC risk (Figure 1).

## 2. Cigarette Smoking

Cigarette smoking has long been known as a predisposing factor for many cancers, including PDAC. Compared with other known smoking-related cancers, cancers of the pancreas, urinary bladder, colon, and rectum have similar relative risks associated with tobacco use (pancreas, 2.0–4.0; urinary bladder, 3.0; colon and rectum, 1.9–2.5) [8]. Meanwhile the average tobacco-associated lung cancer relative risk ranges from 15.0 to 30.0.

Studies show that current cigarette smoking is associated with a doubled risk of PDAC [9]. According to the analysis, this risk increases with the number of cigarettes smoked and the duration of smoking [1]. A recent study found that considering the number of cigarettes smoked, the difference in risk was significant only for those smoking more than 10 cigarettes a day [10]. This 10-year follow-up evaluated 604 cases of PDAC and proved that the risk for current smokers is higher for those under age 65 (19.0%; 95% confidence interval (Cl), 8.1–28.6%) than for older smokers (6.6%; 95% CI, 1.9–11.1%; *p* = 0.030). The same study showed that for current smokers, men have a higher risk of developing PDAC (23.9%; 95% CI, 13.3–33.3%) than women (7.2%; 95% CI, −0.4% to 14.2%; *p* = 0.007), but this may be explained by the fact they usually smoke more cigarettes per day.

In another recent study, the pooled analysis of 10 population-based cohort studies in the Japanese population was conducted. A total of 354,154 healthy subjects and 1779 PDAC cases were analyzed. In this study, on the other hand, among former smokers and ever smokers with 20 pack-years, a significant increase in PDAC risk only in women was noticed [adjusted hazard ratio (aHR) 1.77; 95% CI, 1.19–2.62]. For male former smokers, no significant association was observed (aHR 1.10; 95% CI, 0.89–1.36) [11]. According to this study, the beneficial impact of smoking cessation on PDAC risk appears to be more evident in men than in women.

Regarding smoking among men, confirmation of this relationship was also confirmed in a recent study involving 121,408 Korean men [12]. The study evaluated 245 cases of PDAC. Patients were divided into four groups according to the number of pack-years. Group 1—involved never-smokers, group 2—less than 20 pack-years smokers, group 3—20–40 pack-years smokers, and group 4—more than 40 pack-years smokers. As a result, the multivariate adjusted HR (95% CI) for PDAC in groups 2, 3, and 4 was 1.05, 1.28 and 1.57, respectively (*p* for trend = 0.025). For former smokers, HR (95% CI) demonstrated no statistically significant association in the multivariate adjusted model, indicating a positive effect of smoking cessation.

The risk of PDAC only reaches the level of never-smokers about 20 years after quitting [9]. It was also noticed that smokers are diagnosed with PDAC at an earlier age than non-smokers [13]. The 29,239 confirmed cases of PDAC were evaluated using data analysis from IMPAC Medical Registry Services, Cancer Information Resource File, and 820 datapoints from the University of Michigan Pancreatic Cancer Registry. It was noted that current smokers were diagnosed at a significantly earlier age than non-smoking patients—8.3 years and 6.3 years earlier, respectively, to both centers. Additionally, for the first mentioned database, the age at PDAC diagnosis in current smokers was 2.1 years younger compared to non-smokers (*p* < 0.001).

Other analysis from eight cohorts includes participants primarily of European decent [14]. It was noted that PDAC risk increased significantly with greater intensity (≥30 cigarettes/day: OR = 1.75, 95% CI: 1.27, 2.42), duration (≥50 years: OR = 2.13, 95% CI: 1.25, 3.62), and cumulative smoking dose (≥40 pack-years: OR = 1.78, 95% CI: 1.35, 2.34). Furthermore, higher risk was suggested for total exposure at lower intensity but for longer duration than for smoking at higher intensity for shorter duration. This implies that smoking has a late-stage effect on pancreatic carcinogenesis.

Another important observation is the effect of passive smoking on the increased risk of PDAC. It has been proven that the risk of PDAC was increased for non-smokers, since they are exposed daily to environmental smoking for many hours throughout childhood and are exposed to tobacco smoke at home or at work [15]. There are also reports of negative effects of maternal smoking on the fetus and during early childhood, potentially leading to PDAC in the future [16]. Analyzing a group of 1076 PDAC cases in a hospital-based case-control study, maternal smoking was associated with a statistically significant increase in PDAC risk (Relative Risk (RR), 1.56; 95% CI, 1.13–1.98; *p* = 0.018), which was not observed for the smoking father. Considering never-active smokers reporting their mothers’ smoking, they had increased risk of PDAC; however, the association was not statistically significant.

Despite a range of evidence supporting the contribution of smoking to PDAC, the exact underlying mechanism remains unclear [17]. Generally, as in all cancers, chemicals from cigarettes cause DNA and tumor-suppressor DNA damage and inhibit DNA repair. The smoking-induced build-up of DNA damage in the same cell over time leads to cancer.

In PDAC, one of the smoking-related causes considered may be an increase in fibrosis of the pancreas, which is a typical feature of chronic pancreatitis that is also considered as one of the main risk factors for the development of PDAC [18]. Pancreatic stellate cells and PDAC cells were found to stimulate each other’s proliferation and migration in vitro [19]. Cigarette smoke contains Ahr (Aryl Hydrocarbon Receptor Ligands) ligands that enhance pancreatic fibrosis processes [20].

Interestingly, smoking may indirectly contribute to PDAC through diabetogenic effects manifested by decreased insulin secretion and increased glucagon production in the pancreas, as demonstrated in a study in rats [21]. This diabetic effect may be important, since long-term diabetes has been associated with a 1.5- to 2.0-fold increase in the risk of PDAC, according to epidemiological studies [22].

There are also observations of mechanisms at the molecular level. It has been shown that oncogenic primary microRNA-25 in pancreatic ductal epithelial cells can be over-matured by cigarette smoke through increased N6-methyladenosine modification mediated by NF-κB-associated protein (NKAP). Mature miR-25 in subsequent processes leads to activation of oncogenic AKT-p70S6K signaling, provoking malignant phenotypes of PDAC cells. High levels of miR-25-3p are detected in surgically removed non-tumor pancreatic tissue samples of smokers and in PDAC specimens collected at the time of surgery [23].

The search for a mechanism at the genetic level was undertaken in a large genome-wide interaction study identifying evidence for interaction between smoking and multiple SNPs located in a region on chromosome 2q21.2 on PDAC risk. They examined data on quantitative expression trait loci from the Genotype-Tissue Expression Project and then performed co-localization analysis to determine whether there is support for common SNPs underlying the observed association. This genome-wide analysis of 7937 PDAC cases and 11,774 controls found regions with a qualitative interaction for PDAC by cigarette smoking status at chr2q21.3 in intron 5 of the transmembrane protein 163 (TMEM163) and upstream of the cyclin T2 (CCNT2) [24]. The co-localization results and expression quantitative trait loci signals (QTLs) for TMEM163 and CCNT2 demonstrate the importance of this gene region.

Another study reported the effects of nicotine-derived 4-(methylnitrosamino)-1-(3-pyridyl)-1-butanone (NNK), which is thought to contribute to the development of cancers among smokers, including PDAC [25]. Western blot analysis of HPDE6-c7 cells (developed from normal pancreatic duct epithelial cells) treated with NNK showed increased phosphorylation of tyrosine 992, 1068, and 1173 sites in EGF receptor (EGFR), contributing to cell hyperproliferation and tumorigenesis. Phosphorylation of all three sites was blocked by propranolol, demonstrating the interplay between b-adrenergic receptors and EGFR. This interaction could potentially explain EGFR transactivation, increased phosphorylation, and subsequent activation of the ERK1/2 MAPK cascade. In addition, EGFR is often overexpressed in PDAC cells, so its additional overactivation may be important in this malignancy.

The validity of encouraging patients to quit smoking is evident, but it remains difficult. Current reports suggest a potential for reducing the harm caused by smoking with heat-not-burn (HNB) devices, which deliver a smaller amount of burned substances into the body by heating the tobacco, although more data are necessary to make the convincing recommendations in this issue [26]. Most alarming is the significant increase in the number of young people smoking e-cigarettes. According to the latest 2018 data, the National Youth Tobacco Survey noted that 20.8% of high school students and 4.9% of middle school students currently used e-cigarettes (defined as using an e-cigarette at least 1 day in the past 30 days) [27]. Meanwhile, adult users of e-cigarettes tend to be previous users of combustible tobacco products such as traditional cigarettes [28].

One study evaluated the amount of ROS, carbonyl compounds, and nicotine in a line of heated tobacco products called IQOS [29]. IQOS and combustible cigarettes were found to emit similar amounts of total and free base nicotine. The researchers found that using IQOS in amounts equivalent to a pack a day increased exposure to inhaled formaldehyde and total ROS, respectively, by about twice as much as breathing city air alone. Exposure to acetaldehyde would be more than 100 times greater than when breathing city air. Conversely, smoking a pack a day with IQOS instead of combustible cigarettes would be associated with a 70% and 65% reduction in daily formaldehyde and acetaldehyde intake, respectively, and an 85% reduction in ROS. However, a study of the effects of HNB products on mice lungs demonstrated that even a reduced number of dangerous substances, as tar, carbonyls, VOC, CO, free radicals, or nitrosamines reaching the lungs and lower levels of nicotine are enough to initiate pro-inflammatory reactions which promote PDAC carcinogenesis [30]. Moazed et al., based on studies from the USA and Japan, evaluated healthy adults who had smoked a minimum of 10 conventional cigarettes per day for the past 3 years. Participants in the study were divided into three groups (continued smokers, those who quit, and those who switched to IQOS) and were followed for 90 days. There was a reduction in white blood cell (WBC) levels compared to the cigarette smoking group in the Japanese study, but the other parameters tested, i.e., C-reactive protein (CRP) and forced expiratory volume in one second (FEV1), showed no significant difference between the conventional smokers and the IQOS smoking group in both studies [31].

Vaping devices, which have been on the market longer than HBN, are also worth mentioning. These are battery-powered devices that heat a liquid containing nicotine to the point of vaporization, creating an aerosol. While some evidence is found of fewer negative health effects associated with vaping than with combustible cigarettes, the components of e-liquid and the functions of the device are more varied than those of cigarettes. This may explain reports of aerosol toxicity and its negative effects on respiratory tract cardiovascular outcomes, as well as higher risk of cancers [32]. However, more research on the health effects of these devices is still needed.

HNB devices have been on the market for much too short of a duration to know exactly the cancer risks associated with them, but those studies show that HNBs are not neutral to the body and have negative health effects that are possibly slightly less than conventional cigarettes.

## 3. Alcohol

Alcohol is a frequently considered risk factor of PDAC, but research data are not consistent on this issue. In analyses, confirmation of the link between alcohol consumption and the development of PDAC is usually associated with large volumes of alcohol consumed daily such as in the 2018 study comprising 521,324 participants [33]. According to the results, each 12 g/day of alcohol in men was linearly associated with a 5% increase in PDAC risk for baseline consumption, with a stronger association for alcohol intake greater than 60 g/day. The study also noted a higher risk of PDAC connected to beer and liquors compared to wine consumption. In the women’s group, there was no correlation noted. These differences are likely due to overall lower alcohol consumption among women [34] and, consequently, smaller female study groups in studies. There are also reports of differences in alcohol metabolism in both sexes, but these need to be confirmed. An example is the differential effect of alcohol consumption on insulin sensitivity. After analyzing the results reported by 14 intervention studies, alcohol consumption was only associated with decreased fasting insulin levels and improved insulin sensitivity among women [35].

A dose–response meta-analysis of cohort studies found evidence of a high impact of alcohol consumption, especially liquor, on the risk of developing PDAC. Intake of ≥24 g alcohol per day was associated with an increased risk of PDAC (risk ratio [RR], 1.15; 95% CI: 1.06–1.25) [36]. The moderately increased risk of PDAC with daily high alcohol intake is confirmed by the results of a pooled analysis of case-control studies in The Pancreatic Cancer Case Control Consortium [37].

Alcohol consumption can be considered as an independent risk factor for PDAC, but there are observations assessing the impact of smoking in association with alcohol drinking on PDAC risk. The population risk attributable to smoking increased to 25.7% when considered in combination with alcohol drinking, compared to 13.6% for smoking alone [38].

A nationwide, population-based cohort study of more than nine million people found a J-shaped association between alcohol consumption and PDAC risk in normoglycemic participants, but not in those with impaired fasting glucose and diabetes [39]. The study also highlighted differences in risk according to the frequency of alcohol consumption. Participants with normoglycemia consuming alcohol 1–2 days per week had low risk of PDAC (HR, 0.91; 95% CI, 0.85–0.97), while consuming alcohol for 5 or more days per week had an increased risk of PDAC (HR, 1.13; 95% CI, 1.002–1.27).

Another case-control study in Taiwan did not confirm the increase of PDAC risk associated with alcohol use. A gene–environment interaction between alcohol use and polymorphisms of two ethanol-metabolizing genes, ADH1B and ALDH2, on PDAC risk was evaluated. No significant PDAC risk increase was noted even in those genetically susceptible to the carcinogenic effects of alcohol, i.e., carriers of ADH1B*2/*2 (fast activity) combined with ALDH2*1/*2 (slow activity) or ALDH2*2/*2 (almost non-functional). However, this may be explained by the low number of heavy drinkers (defined as drinking more than 42 g of pure alcohol for women and more than 48 g for men) in the study, because only 7.2% of cases and 4.1% of controls met this definition [40].

Another study comprising 196 patients with PDAC from 4 North Vietnamese hospitals noted the stronger association between chronic pancreatitis and PDAC than between alcohol consumption and PDAC [41]. The odds ratio (OR) (95% CI) was 2.21 (1.42–3.45) for inflammatory disease and 1.48 (0.91–2.42) for current alcohol consumption. In this case, alcohol consumption intake of at least 30 g/day or 150 g/week was considered.

However, it is worth highlighting the indirect effect in which alcohol can contribute to the increased risk of PDAC. Namely, heavy alcohol drinking has been positively associated with risk of chronic pancreatitis [42] and non-insulin dependent diabetes mellitus [43]—two long known diseases associated with an increased risk of PDAC [44].

The carcinogenicity of alcohol may derive from the toxic effects of nonoxidative fatty acid metabolites, leading to an increase in calcium ions, which mediate toxicity in the pancreatic acinar cell [45]. The disproportion between the activity of ADH (alcohol dehydrogenase) and ALDH (aldehyde dehydrogenase)—enzymes involved in the elimination of ethanol, noted in alcohol-induced tumors—may also play an important role. These disorders may be due to polymorphisms in genes associated with ADH and ALDH [46]. Thus, in an analysis of the combined effect of alcohol consumption and genotype, a significant effect of alcohol was observed in individuals with the ALDH2 Lys+, ADH1B His/His or ADH1C Arg/Arg allele (trend-P = 0.077, 0.003, or 0.020, respectively), each of which is associated with high concentrations or a rapid production of acetaldehyde [47].

Moreover, alcohol consumption is associated with increased production of ROS, which are known to promote carcinogenesis. The new study observed that in PDAC, increased levels of ROS can lead to increased metastasis through ERK activation [48]. There are many mechanisms leading to the production of ROS with ethanol consumption, among others: CYP2E1 activation. ROS produced by CYP2E1 result in the accumulation of lipid peroxidation products that can react with certain DNA bases to form highly mutagenic adducts [49]. ROS accumulation leads to upregulation of vascular endothelial growth factor (VEGF) and monocyte chemotactic protein (MCP-1), which promote angiogenesis and tumor metastasis [50].

Alcoholics also have lower levels of retinoids, which are known to promote cell differentiation and apoptosis [51]. Moreover, alcohol impairs the innate immune response by suppressing NK cell function, activating monocytes and macrophages to produce pro-inflammatory cytokines such as TNF-α, interleukins IL-1 and IL-6, and chemokine IL-8, It also causes dendritic cell abnormalities by disrupting their phagocytic functions [45]. These changes may increase the inflammation process and reduce the capacity of immune response to identify and kill atypic cells and, thereby, facilitate tumorigenesis in the pancreas.

In addition, the adaptive immune response is altered due to alcohol consumption by, among others, the upregulation of certain T-lymphocyte subpopulations. In a mouse study, a 201T human lung adenocarcinoma cell line was implanted into the lungs of ethanol-fed BALB/c mice, which also received an anti-CD4 antibody. Compared to control mice, the ethanol-fed mice had larger tumor sizes [52].

## 4. Chronic Pancreatitis

Chronic pancreatitis (CP) may seem unrelated to environmental risk factors for PDAC, but the disease is worth considering because of the factors that lead to its development. These factors are mainly alcohol and cigarette smoking, which, in combination, predispose even stronger to the development of CP [53]. The risk of developing PDAC in patients with CP may be dependent on CP duration [54]. In the recent study, the increase in PDAC risk caused by CP is maintained for about 10 years after CP diagnosis, and after that time, it begins to decline [55]. After identifying 13 studies, the pooled effect estimate (EE) for PDAC in patients with chronic pancreatitis was 16.16 (95% CI: 12.59–20.73), referring to patients whose PDAC was diagnosed up to 2 years after the diagnosis of chronic pancreatitis. This risk decreased when the cancer was diagnosed after an extended period of up to 5 years (EE: 7.90; 95% CI: 4.26–14.66) or a minimum of 9 years (EE: 3.53; 95% CI: 1.69–7.38). Another meta-analysis of 12 studies on chronic pancreatitis showed an increased risk of PDAC in patients with CP (SIR: 22.61, 95% confidence interval [CI]: 14.42–35.44), and the incidence of PDAC was increasing with the duration of CP disease. In addition, the increased risk was maintained after excluding potentially incident cases in which PDAC was diagnosed in close temporal proximity to CP diagnosis [56]. These findings suggest the validity of attentive monitoring of patients with long-term CP for the development of PDAC.

A cohort study compared the risk of PDAC in four groups: one episode of unspecified pancreatitis, one episode of acute pancreatitis (AP), recurrent pancreatitis, and, finally, chronic pancreatitis. The SIR for all cohorts combined was 2.8 (95% CI, 2.5–3.2), similarly for both sexes. The highest risk was observed in patients with chronic pancreatitis (SIR, 7.6; 95% CI, 6.0–9.7) and with one attack of unspecified pancreatitis (SIR, 7.3; 95% CI, 3.5–13.4). Interestingly, in the subcohorts, a significantly increased risk after 10 or more years was associated only with recurrent acute or unspecified pancreatitis (SIR, 2.2; 95% CI, 1.2–3.7) [57]. The 2023 retrospective study comprising 16,475 PDAC cases presents an interesting finding, showing the risk of PDAC for groups of patients with acute pancreatitis without (AP group) and with underlying CP (APCP group) and those with CP alone (CP group). Due to the result, the PDAC risk was significantly higher in APCP group than in either AP (adjusted HR 2.24, 95% CI 1.69–2.96, *p* < 0.001, APCP vs. AP) or CP patient groups (adjusted HR 1.94, 95% CI 1.39–2.71, *p* < 0.001, APCP vs. CP). Thus, the study suggests that the risk of PDAC increases with the number of AP episodes and is additive to the higher risk of PDAC due to CP [58].

All types of chronic pancreatitis, including the one caused by environmental factors, predispose to PDAC [59]. In both pancreatitis and PDAC, a response to proinflammatory cytokines is observed in the form of proliferation of acinar, ductal, and stellate cells with epithelial–mesenchymal transition and further-progressive carcinogenesis [60]. Secondary pro-inflammatory mediators induced by cytokines are chemokines, which are also known to promote tumorigenesis, proliferation, metastasis, and angiogenesis of various cancers [61]. Expression of the pro-inflammatory cytokine TNF-α is elevated in pancreatic ductal adenocarcinoma initiation phase and also predicts poor survival of PDAC patients. Moreover, anti-TNF-α in vivo treatments showed effects in reducing desmoplasia and the tumor promoting inflammatory microenvironment in PDAC [62]. A mouse model of PDAC xenograft was used, and mice were divided into four groups using different treatments in each group. Compared to the saline and chemotherapy group, the combination of chemotherapy and anti-TNF-α treatment showed approximately 20 and 10 days longer survival times, respectively. Furthermore, in pancreatitis and PDAC, acinar cells can differentiate into ductal cells, which is driven by the inflammatory transcription factor NFATc4, which also initiates PDAC by direct transcriptional induction of Sox9 [63]. Together with KRAS mutations and loss of suppressor barriers p16/INK4A/CDKN2A, TP53, and SMAD4/DPC4, these inflammatory reactions contribute to pancreatic carcinogenesis [62].

There are new findings on therapy in chronic pancreatitis. IL-15 plays an anti-inflammatory role, but it has also been proven that Il-15 administration promotes cytotoxicity by activation of NK cells, which have reduced activity in PDAC patients [64]. In addition, CXCL4 levels are elevated in mild and severe acute pancreatitis, which directs CXCL4 inhibition as a therapy to treat pancreatitis. Administration of a monoclonal antibody (mAb) against CXCL4L1 blocked the growth of CXCR3-positive tumors, a receptor for CXCL4, and inhibited the growth of pancreatic ductal adenocarcinoma through antiangiogenic function [65]. Thus, targeted therapies aimed at controlling pro-inflammatory cytokines and chemokines, inhibiting immune cell infiltration and abnormal inflammatory signals, and increasing T-cell activation may offer prospects for potentially reducing the incidence of PDAC through early intervention [60].

## 5. Viral Hepatitis

Hepatitis B virus (HBV) and hepatitis C virus (HCV) are well-known oncogenic viruses that are mainly associated with liver damage, but there are data supporting their involvement in carcinogenesis in other organs, including the pancreas [66].

It has been discovered that various cells of extrahepatic organs can support HCV replication, such as the pancreatic cells. In autopsy tissue samples from nine patients with hepatitis C, HCV RNA and HCV antigens were found in pancreas acinar cells and pancreatic duct epithelial cells of [67]. There are suggestions of a mechanism underlying the presence of hepatotropic viruses in the pancreatic tissues. The liver and pancreas lie in anatomical proximity, with their bile ducts and blood vessels in contact with each other, potentially providing open pathways for microorganisms [68]. Moreover, HBV-DNA integration with Southern blot was also observed in pancreatic acinar cells and in liver metastases of PDAC in patients infected with HBV [69]. In one of the two patients studied, intense viral proliferation was noted in the liver, both within PDAC metastases, and in non-tumor tissues. It was suggested that virus proliferation may have taken place in pancreatic cells.

Another observation suggesting the contribution of HBV viruses to PDAC pathogenesis is that acute pancreatitis may be an extrahepatic manifestation of viral hepatitis [70]. The study showed that acute exacerbation of chronic HBV infection was responsible for 5.6% of acute pancreatitis in the Chinese population. It can be explained by two mechanisms: increase in viral antigen that may become toxic to the pancreatic cells or the immune response against HBV-infected hepatocytes that may also be directed against HBV-infected pancreatic cells. A history of acute pancreatitis is also associated with a higher risk of developing PDAC [71].

A controlled study analyzing 476 PDAC cases, including 876 subjects, showed that previous exposure to HBV may be associated with the development of PDAC [72]. Blood samples were tested for antibodies to HBV core antigen (anti-HBc) and antibodies to HBsAg (anti-HBs), which showed that past recovered exposure to HBV (anti-HBc-positive) or after acquiring immunity (anti-HBs-positive) was associated with highly increased risk of PDAC (adjusted odds ratio (AOR), 2.3; *p* = 0.01). In addition, this study suggests the possibility of HBV reactivation during patient’s chemotherapy.

In a 2021 prospective study evaluating the Chinese population, 93,402 Chinese participants with hepatitis B were monitored for about 13 years. A total of 1791 cases of gastrointestinal cancer were identified, including 167 cases of PDAC. Other cancers detected included liver, stomach, gallbladder or extrahepatic bile ducts, small intestine, esophagus, large intestine, and pancreas. The direct association between hepatitis B and the occurrence of PDAC was demonstrated [(HR) = 1.86, 95% confidence interval (CI): 1.10–3.99] [73].

Moreover, another recent Chinese study proved that HBV vaccination may reduce the risk of pancreatic malignancies [74]. A total of 4748 patients comprising seven different cancers including 340 patients with PDAC and 57,499 controls were included. It was noted that anti-HBs represented the protective factor for PDAC (aOR = 0.58, 95% CI: 0.42–0.82).

The presence of anti-HBs is usually associated with immunization after vaccination. Thus, it seems that hepatotropic virus infection prevention may also contribute to lower PDAC incidence. For that reason, research data are mainly focused on the Asian ethnicity and, in particular, on two high HBV endemic regions, China and Taiwan, where HBsAg prevalence is over 7–8% compared to the USA, where the global HBsAg prevalence is less than 2% [75]. Nevertheless, there are emerging single scientific studies, for instance, from Sweden. In population-based studies that were performed on patients with HBV infection, they had an increased risk of hepatocellular carcinoma and other cancers such as PDAC (standardized incidence ratio 1.79 (95% CI 0.85–3.30)). Increased risk of PDAC concerns HBV patients who were infected at a younger age (i.e., <30 years at diagnosis of HBV) [76]. Another meta-analysis compared the increased risk of developing PDAC due to HBV infection by study region. The summary OR for Asian patients was 1.300 (95% CI: 1.057–1.600, *p* = 0.013) for HBV-positive patients, and the OR for non-Asian patients was 1.862 (95% CI: 1.0063–3.260, *p* = 0.030) [77].

## 6. Physical Activity

Physical activity has always been known for its beneficial effects on the body, including reducing the risk of developing cancer. Therefore, the latest WHO recommendations advise 150–300 min of moderate-intensity physical activity per week (refers to the physical activity that is performed between 3 and <6 times the intensity of rest (METs)), or 75–150 min of vigorous-intensity physical activity for adults per week (refers to the physical activity that is performed at >3 METs (i.e., >3 times the intensity of rest)) and emphasize that a little activity is better than none [78]. A prospective study of 95,962 participants found that greater adherence to the 2018 World Cancer Research Fund/American Institute for Cancer Research (WCRF/AICR) recommendations was associated with a lower risk of PDAC. A higher overall WCRF/AICR score was found to be associated with a reduced risk of PDAC (hazard ratio (tertile 3 vs. 1):0.67; 95% confidence interval: 0.49, 0.90; *p*(trend) = 0.0099). The WCRF/AICR overall score was the sum of the points assigned to each recommendation, ranging from zero to eight points. Higher scores indicated a greater compliance with the WCRF/AICR guidelines. Among the eight components of the total WCRF/AICR score, only compliance with “be physically active” was linked to a reduced risk of PDAC [79]. In the study, the physical activity level referred to the total time of moderate to vigorous activity per week.

A comprehensive systematic review and meta-analysis found that physical activity is not strongly associated with PDAC risk, and additionally, the association is not modified by smoking status or BMI level [80]. However, a potential reduction in PDAC risk was noted for consistent physical activity over time (RR 0.86, 95% CI 0.76–0.97) compared with recent past physical activity (RR 0.95, 95% CI 0.90–1.01) or distant past physical activity (RR 0.95, 95% CI 0.79–1.15, p-difference by timing in life of physical activity = 0.36). Consistent physical activity in this study meant physical activity maintained for more than 10 years, as well as occupational physical activity. Recent physical activity referred to physical activity up to 3 years prior to the baseline questionnaire or up to 3 years prior to the time of first appearance of symptoms/diagnosis/interview. Distant past physical activity was defined as physical activity three or more years prior to PDAC diagnosis.

Physical activity may lead to a reduced risk of PDAC indirectly by reducing BMI, but also as an independent factor [81]. In a prospective study in participants younger than 60 years, higher physical activity was associated with decreased PDAC risk (highest vs. lowest category HR = 0.27, 95% CI = 0.07–0.99). Interestingly, the association remained even when BMI was included in the analyses.

## 7. Occupational Exposure and Physical Factors

There are cancers known to be strongly associated with environmental factors present in the workplace [82]. The data are accumulating on the effects of chemicals on the development of PDAC, including exposure to pesticides, asbestos, benzene, and chlorinated hydrocarbons [83]. The increased risk is particularly apparent for pesticides such as fungicides and herbicides. [84]. It is thought that these substances can reach the pancreas through the bloodstream or via the bile reflux and thereby exert genotoxic effects, including altered methylation, activation of oncogenes, inactivation of tumor suppressor proteins, and formation of DNA adducts [83]. There are also suggestions of a link between chromium and nickel and the development of PDAC [85].

A Spanish study involving 118 cases of PDAC and 399 controls determined levels of 12 trace elements in nail samples by inductively coupled plasma mass spectrometry. The study found a correlation between high concentrations of chemical elements such as cadmium (OR = 3.58, 95% CI 1.86–6.88; p(trend) = 5 × 10^−6^) and arsenic (OR = 2.02, 95% CI 1.08–3.78; p(trend) = 0.009), and the development of PDAC [86]. In contrast, high concentrations of selenium (OR = 0.05, 95% CI 0.02–0.15; p(trend) = 8 × 10^−11^) and nickel (OR = 0.27, 95% CI 0.12–0.59; p(trend) = 2 × 10^−4^) were inversely associated with PDAC risk. Cadmium was found to cause pancreatic cell transdifferentiation, inhibit DNA repair, and induce or regulate the activity of several oncogenes or tumor-suppressor proteins that are expressed in human PDAC [87]. Arsenic may contribute to carcinogenesis by inducing oxidative stress, which causes DNA strand breaks, alkali-inducible sites, and, ultimately, DNA adducts [88].

A recent 2021 case-control study in the cohort of workers in poultry plants workers showed increased risk of PDAC mortality, especially in chicken and turkey plants [89]. It may be caused by the exposure to zoonotic oncogenic viruses, against which high serum levels antibodies were detected in the study group [90].

Another study examined the dose–response relationship between duration of occupational exposure to chemical agents and PDAC risk using a meta-regression and a meta-analysis. All types of industries and chemical agents in 288,389 participants were included. The risk of developing PDAC increased with exposure duration of 1–10 years (relative risk [RR] = 1.04; 95% CI 1.02–1.06), 11–20 years (RR = 1.11; 95% CI 1.05–1.16), and 21–30 years (RR = 1.39; 95% CI 1.12–1.73). Regarding the type of industry, the RR of PDAC were higher especially for the chemical, metal, and plastics and rubber industries. Regarding the type of chemical agent, the risk was especially higher for ethylene oxide and PAHs [91].

Another finding is the effect of light at night (LAN) on PDAC risk. A recent epidemiological study involving 464,371 participants and 2502 PDAC cases showed that people living in an area with a lower LAN quintile had a 27% increased risk of developing PDAC [HR (95% CI), 1.24 (1.03–1.49)] [92]. LAN was estimated through satellite imagery at baseline (1996), and incident PDAC cases were identified from state cancer registries. Annual composite LAN measures were obtained from the Operational Linescan System archive of the U.S. Defense Meteorological Satellite Program, which was run by the National Oceanic and Atmospheric Administration’s Earth Observation Group. LAN measures were converted to units of radiance (nanowatts/cm^2^/sterradian(sr)).

Relevant to the correlation of circadian rhythm with tumorigenesis may be genes such as Per1, Per2, Cry1, Cry2, and Bmal1 which code for the proteins regulating circadian rhythms. The disruption of this process can lead to dysregulation of cell proliferation and, consequently, tumorigenesis [93]. These genes also play a role in regulating cell cycle progression, DNA damage response, and genome stability. Through RT-PCR methods, several genes related to circadian rhythm were found to be under-expressed in PDAC [94]. One of the five genes that were most strongly under-expressed was the Drosophila protein homolog 1 (PER1) gene. Per1 is one of the eight known core circadian rhythm regulators. The four remaining genes are CDC37, CDNA FLJ42412 fis, clone BLADE2001138, which is highly similar to CTRA_GADMO Chymotrypsin A, REG1a, and PRSS2.

## 8. Microbiome

The data are accumulating on the role of microbial dysbiosis in oncogenesis [95]. A study uncovered the existence of a distinct gut microbiome that contributed to the progression of mouse pancreatic oncogenesis [96]. They demonstrated the presence of the own microbiome in the pancreas, which is linked to the stage of the disease in mice and humans. The pancreatic internal microbiome in PDAC tumors from 12 patients was characterized using 16S rRNA gene sequencing. The bacteria detected were predominantly Proteobacteria (45%), Bacteroidetes (31%), and Firmicutes (22%). The distinct bacterial dysbiosis associated with PDAC has been shown to cause both innate and adaptive immune suppression. Removal of the gut microbiome led to reduced infiltration of myeloid-derived suppressor cells (MDSCs) and reprogramming of tumor-associated macrophages (TAMs) into an M1-like, tumor-protective phenotype. This may be the explanation underlying the result of slower progression of carcinogenesis after oral antibiotic therapy in WT (wild type) mice in the study. To establish access of intestinal bacteria to the pancreas, fluorescently labeled Enterococcus faecalis and Escherichia coli were administered orally. In both cases, the bacteria migrated into the pancreas, suggesting that gut bacteria can directly affect the pancreatic microenvironment.

There are three mechanisms that may explain the aforementioned link [97]. The microbiome species may lead to inflammation, inhibit interactions between macrophages and T cells, and promote polarization of the T-cell response to Th2. A second mechanism may be the effect of the microbiome on the immune system, and a study of transplanting human fecal microbiota into mice from donors with short-term survival (STS), long-term survival (LTS), or control donors found that modulation of the tumor microbiome can affect tumor growth, but also immune infiltration of the tumor [98]. Using 16S rRNA gene sequencing, they analyzed the composition of the tumor microbiome in the aforementioned PDAC patient groups and found the signature of three tumor bacterial species: *Saccharopolyspora* sp., *Pseudoxanthomonas* sp., *Streptomyces* sp. to be significantly enriched in LTS patients compared to STS patients. There was also a higher density of CD8+ T cells in LTS compared to STS patients (*p* = 0.008) in the validation cohort. After clustering the tissue density of CD8+ T cells with bacterial species enriched in LTS patients, a positive Spearman correlation was found between the two variables (*p* < 0.0001, *p* = 0.006, and *p* < 0.0001, respectively), suggesting that the diversity of the tumor microbiome and the presence of these three bacterial genera in the tumor may contribute to the anti-tumor immune response by promoting CD8+ T cell recruitment and activation.

Another mechanism indirectly linked to the development of PDAC may be microbiome-induced metabolic disturbances that lead to two known risk factors for PDAC: obesity and diabetes. A study found that body mass indices of lean mice can be increased by transplanting the gut microbiome from obese animals [99]. It was shown that in diabetes, the ratio of Firmicutes to Bacteroidetes is increased, which affects carbohydrate metabolism and the production of SCFAs [100]. There is an increased production of acetate, which leads to insulin resistance and increases the production of ghrelin (appetite stimulating hormone) in the stomach. The production of butyrate decreases, which also indirectly promotes insulin resistance.

In addition, oral bacteria have recently been considered to contribute to carcinogenesis [101]. In 1 prospective cohort study, serum bacterial antibody levels in 405 PDAC patients and 416 matched controls were evaluated [102]. It found that those with high levels of antibodies to the pathological periodontal bacterium Porphyromonas gingivalis ATTC 53,978 had a twofold higher risk of PDAC. In the same study, the number of antibodies against oral commensal bacteria was analyzed, and it was found that people with higher levels of antibodies had a 45% lower risk of PDAC, which may be related to the inhibition of pathogenic species growth. Another meta-analysis observed that periodontal disease increased the relative risk of PDAC by 1.74 times, and that the risk was additionally increased by 1.54 times for patients when teeth were missing [103]. A more recent study involving 1,352,256 participants noted exactly the same observation. In subgroup analyses, those with periodontal disease (HR, 1.38; 95% CI, 1.12–1.71) had a higher risk of developing PDAC than those with tooth loss (HR, 1.19; 95% CI, 0.97–1.46) [104]. Preventing and treating periodontitis appears to be an important and easily achievable preventive measure.

The new study described the characterization of the oral and intestinal microbiota of the group of 40 patients with PDAC in parallel using 16S rRNA analysis [105]. Oral pathogenic genera (Granulicatella, Peptostreptococcus, Alloprevotella, Veillonella, Solobacterium, Streptococcus, and others) showed significant enrichment in PDAC patients. In the intestine, probiotics such as Bifidobacterium and Butyricicoccus were reduced, while opportunistic species (Prevotella, Escherichia-Shigella, Peptostreptococcus, Actinomyces, etc.) were significantly enriched.

The effect of human microbiome on PDAC development still requires additional research, but the results so far indicate that the relation is strong.

## 9. Obesity and Diet

Obesity, defined as a BMI of 30 or more [106], is known to predispose to the occurrence of many cancers, including PDAC [107].

In one meta-analysis, obesity was proven to contribute to PDAC with the risk ratio 1.36 for men and 1.34 for women [108]. A study with a pooled analysis of 14 cohort studies involving 846,340 people found that obese people had a 47% higher risk of PDAC compared to individuals with a body mass index (BMI) at baseline between 21 and 22.9 kg/m^2^ (95% CI: 23–75%) [109].

A group of 642 overweight individuals (BMI 25–29.9) aged 14 to 39 years had an increased risk of PDAC (highest odds ratio [OR], 1.67; 95% confidence interval [CI], 1.20–2.34) [110]. Moreover, in this case-control study, the median age of onset of PDAC was 64 years for patients with regular weight, 61 years for overweight patients [*p* = 0.02], and 59 years for obese patients [*p* < 0.001]). In addition, the strongest correlation was noted for young adults. It was observed that overweightness starting at age 14–19 or 20–29 and obesity starting at age 20–29 or 30–39 demonstrated the strongest association with PDAC risk. Another meta-analysis involving 3,495,981 people and 8062 PDAC cases confirmed this positive association between BMI and PDAC risk in both sexes. A 5 kg/m^2^ increase in BMI resulted in a 12% PDAC risk increase [111].

The mechanisms of the relationship between BMI and PDAC are complex. Fat accumulation induces hypoxia and mild inflammation in adipose tissue, resulting in increased secretion of TNF-alpha, IL-1 β, and IL-6 [112]. These contribute to tumorigenesis via NF-kB, MAPK/ERK and JNK signaling pathways. Elevated serum leptin levels may promote pancreatic tumor invasion and metastasis through activation of the JAK2/STAT3 axis. Adipokines (including resistin, lipocalin-2, apelin, and wisfatin), which stimulate PDAC tumorigenesis, are also secreted. Decreased release of adiponectin by dysfunctional adipocytes reduces its anti-cancer effects, mediated by the JAK2/STAT3 axis. The study examined the association between prediagnostic plasma adiponectin and subsequent risk of PDAC. Plasma adiponectin was inversely associated with PDAC risk, which was consistent across the five prospective cohorts (*P* heterogeneity = 0.49) and independent of other markers of insulin resistance (e.g., diabetes, body mass index, physical activity, plasma C-peptide) [113].

A recent study found abnormal beta cell expression of the peptide hormone cholecystokinin (CCK) in response to obesity and demonstrated that it promotes oncogenic Kras-driven pancreatic ductal tumorigenesis [114]. Cck expression of islets was evaluated by IHC (immunohistochemistry) in ob/ob JAK mice. Cck expression was increased in islets from obese models and markedly reduced by weight loss interventions. Afterwards, islets from human donors without known malignancy were obtained, and a positive association between BMI and CCK expression was observed. Subsequently, transgenic MIP-Cck mice which express beta cell-specific Cck comparable to ob/ob mice were used. Transgenic mice nonobese (MKC:MIP-Cck; Pdx1-Cre; KrasLSL-G12D/WT) displayed a significant increase in tumor burden compared to KC controls, supporting the hypothesis that islet Cck overexpression may function as an independent driver of pancreatic ductal tumorigenesis.

In obesity, there are alterations in the microbiota content, caused by the unfavorable diet [115], contributing to the PDAC development.

Many reports suggest that red meat consumption increases the risk of PDAC [116,117]. A metanalysis reviewing 11 prospective studies involving 6643 PDAC patients found that a 50 g daily increase in processed meat consumption was associated with a 19% increase in PDAC risk [118]. On the other hand, the Netherlands Cohort Study involving 350 cases of PDAC [119] evaluated the association of meat consumption, and also studied fish, eggs, total dietary fat, and different types of fat and PDAC occurrence. No positive association between high intake of these products and PDAC incidence was noted. Intake amounts were assessed by a validated 150-item food frequency questionnaire.

A Multi-Center Case-Control Study in China examining 323 cases showed that high meat consumption was associated with a higher risk of PDAC (OR = 0.59 for consumption 1–2 times/week vs. more than 3 times/week; 95% CI: 0.35–0.97). In contrast, a protective effect was observed for fruit consumption (OR = 1.73 for consumption 1–2 times/week vs. more than 3 times/week; 95% CI: 1.05–2.86) [120]. Another randomized controlled trial yielded a positive association between nitrite from processed meat and PDAC, but no association with red meat intake, which highlights the role of nitrates themselves and their possible negative effects [121].

A study in mice proved a possible mechanism behind the link between PDAC and obesity. This study suggests that high-fat diet may act as an external trigger to induce inflammation, which then promotes Ras protein activation. High levels of Ras activity are associated with the development of PanINs, subsequently leading to PDAC [122].

Randomized controlled trial in postmenopausal women suggested the potential effect of a low-fat diet on reducing PDAC risk in those with a BMI above 25 (HR = 0.71, 95% CI = 0.53 to 0.96) [123]. The diet in the introduced intervention consisted of the reduced fat intake and increased fruits and vegetables, as well as cereals.

The dietary fiber itself that is present in vegetables also contributes to lower PDAC risk [124]. This systematic review with metanalysis included 18 records. The study investigated the effects of dietary fiber, which is divided into soluble fiber (containing mainly pectin) and insoluble fiber (based mainly on cellulose). The results of the study confirmed that both types of fiber can act synergistically to reduce the risk of PDAC. With both soluble and insoluble fiber included, the OR moved from 0.75 (in the pooled estimate) to 0.62 and 0.58, respectively. The strength of the association increased also when the analysis was grouped only by gender (reduction of around 60% of PDAC risk among women, compared to 30% lower risk among men).

There are reports of other dietary compounds with potential anticancer effects that include: B vitamins, grape seed extract, silibinin (falvonolignan extracted from milk thistle), curcumin, Epigallocatechin 3-Gallate (polyphenol found in tea leaves of Camellia sinensis), and probiotics [125]. The previously mentioned study reported that tea consumption (OR = 0.49; 95% CI: 0.30–0.80) was associated with a half reduction in PDAC risk [120].

## 10. Conclusions

Due to the increasing incidence of PDAC and the constant diagnostic and therapeutic difficulties, there is a necessity to analyze modifiable cancer risk factors and identify high-risk patients. Although the mechanisms leading to the increased impact of these factors still need to be clarified, factors such as cigarette smoking, alcohol consumption, and chronic pancreatitis seem to be the most confirmed factors that have been investigated for the longest time. The latest factor currently drawing the attention of researchers seems to be acute pancreatitis. Observations on the influence of oral and intestinal microbiota and hepatotropic viruses are ongoing, as well as the analysis of vaccination against them as a potential future prevention of PDAC. Many substances, such as chemical and physical compounds associated with the occupational environment, are evaluated as contributing to PDAC, although their independent effects are not clearly evaluated yet.

A clear definition of the PDAC modifiable risk factors has the immediate use to direct the population education and awareness and high-risk patients counselling. In addition, this knowledge may contribute to elaborate upon the additional criteria for the possible target groups for PDAC screening.

## Data Availability

Not applicable.
